# Fulvic Acid Alleviates Paper Sludge Toxicity in Canola (*Brassica napus* L.) by Reducing Cr, Cd, and Pb Uptake

**DOI:** 10.3389/fpls.2022.874723

**Published:** 2022-06-09

**Authors:** Sheza Ayaz Khilji, Zahoor Ahmad Sajid, Sidra Fayyaz, Anis Ali Shah, Adnan Noor Shah, Mamoona Rauf, Muhammad Arif, Seung Hwan Yang, Sajid Fiaz

**Affiliations:** ^1^Division of Science and Technology, Department of Botany, University of Education, Lahore, Pakistan; ^2^Institute of Botany, University of the Punjab, Lahore, Pakistan; ^3^Department of Agricultural Engineering, Khwaja Fareed University of Engineering and Information Technology, Rahim Yar Khan, Pakistan; ^4^Department of Botany, Garden Campus, Abdul Wali Khan University Mardan, Mardan, Pakistan; ^5^Department of Biotechnology, Abdul Wali Khan University, Mardan, Pakistan; ^6^Department of Biotechnology, Chonnam National University, Yeosu, South Korea; ^7^Department of Plant Breeding and Genetics, The University of Haripur, Haripur, Pakistan

**Keywords:** *Brassica napus*, phytostabilization, growth, heavy metal stress, stimulant

## Abstract

Heavy metal toxicity reduces the growth and development of crop plants growing in metal-contaminated regions. Disposal of industrial waste in agricultural areas has negative effects on the physiochemical activities of plants. This research aimed to examine the fulvic acid (FA)-mediated efficacy of *Brassica napus* L. regarding stress tolerance in soil amended with paper sludge (PS). For this purpose, plants were grown for 90 days under greenhouse conditions at various concentrations of PS-amended soils (0, 5, 10, and 15%) being irrigated with water containing FA (0, 10, and 20%). All the physicochemical parameters of PS were carried out before and after plant transplantation. Paper sludge toxicity reduced the growth (shoot/root length, fresh/dry weight of shoot/root, numbers of flowers and leaves) and physicochemical characteristics of exposed *B. napus* plants. In comparison, FA application improved growth by reducing the metal uptake in the shoot of plants grown at various concentrations of PS. An increasing trend in antioxidant enzyme activity was observed by increasing the FA concentration (0%-10% and 20%). Post-harvest analysis indicated that the amount of tested metals was significantly reduced at all PS concentrations. Minimum metal uptake was observed at 0% concentration and maximum at 15% concentration of paper sludge. Additionally, FA application at 20% concentration reduced Chromium (Cr), Cadmium (Cd), and Lead (Pb) uptake in the shoot from 6.08, 34.42, and 20.6 mgkg^−1^ to 3.62, 17.33, and 15.22 mgkg^−^1, respectively. At this concentration of paper sludge in the root, 20% FA reduced Cr, Cd, and Pb uptake from 11.19, 44.11, and 35.5 mgkg^−1^ to 7.88, 27.01, and 24.02 mgkg^−1^, respectively. Thus, FA at 20% concentration was found to be an effective stimulant to mitigate the metal stress in *B. napus* grown in paper sludge-polluted soil by reducing metal uptake and translocation to various plant parts.

## Introduction

Rapid industrial growth has increased pollution levels across the world (Awa and Hadibarata, 2020). Pakistan has been facing increasing air, land, and water pollution, directly affecting human and animal lives. Since no effective strategy has been adopted to address pollution, Pakistan has become one of the most polluted countries in the world (Raza et al., 2017). The toxic emissions from the industries affect the environment in several ways, which led to different diseases in humans and animals, resulting in death in many cases (Bergstra et al., 2019). There are several technologies used to treat and manage industrial waste, such as physical, chemical, and biological, produced by various industries. The landfill process of waste management is used not only in Pakistan but also in other countries of the world to dispose of solid waste. These landfill deposits cause soil pollution by pollutants leaching into the soil (Abou El-Anwar, 2019; Ferronato and Torretta, 2019).

The paper industry forms a major portion of the world's economy in terms of paper consumption by consumers and total wages paid. The total paper consumption in Pakistan is 3.5 kg/year per capita. The paper industry comprises over 100 paper mills, and the consumption capacity is 434,740 tons (Rashid and Hussain, 2014). Nearly 900,000 tons is the present installed capacity of the paper industry. Different raw materials such as wheat straw, grass, bagasse, rice straw, and cotton linter are commonly used to produce paper products in Pakistan. Paper sludge is a semi-solid material produced from the paper mill industries, which is a remarkable source of organic material, several macro/micronutrients, various essential/non-essential metals, and toxic metals such as Pb, Cr, Cd, and Nickel (Ni) (Khilji et al., 2021). The composition of the sludge varies depending on the raw material used in the paper-making process (De-Azevedo et al., 2018). Disposal strategies of paper sludge generated from different industries depend upon the areas and the regulation assent.

*Brassica napus* L. is used as a source of cooking oil and biofuel since it has high contents of oil (>40%) in its seed (Zeremski et al., 2021). It is a hyper-accumulator plant that is used to accumulate heavy metals by employing chelators such as organic acids. Chelators formed complexes like carbonate, sulfate, and phosphate precipitate which are then immobilized in the extracellular/ intracellular spaces (vacuole) (Yan et al., 2020). Another strategy to reduce the heavy metal toxicity is by converting them into less toxic forms or allowing normal concentrations reach plants (Rajkumar et al., 2013; Mourato et al., 2015).

Fulvic acid (FA) acts as a metal chelator in plants. It ensures the easy availability of minerals by converting them into organic compounds. It increases the water storage ability and water infiltration capacity of the soil (Wright and Lenssen, 2013). It helps in enhancing plant tolerance to heavy metal stress. It also improves the antioxidant enzymatic system under heavy metal stress and lowers the heavy metal translocation in different parts of the plants (Yildirim et al., 2021). Moreover, FA increases the vegetative growth of plants by improving nutrient availability, improving the photosynthesis processes, and minimizing the effect of heavy metals (Wang et al., 2019). Fulvic acid reduces readily soluble and exchangeable forms of heavy metals in the contaminated soil but increases their plant-available forms.

This research work was designed to evaluate the effect of FA application to alleviate Cr, Cd, and Pb toxicity in *B. napus* plant grown in soils amended with paper sludge containing the toxic heavy metals. Another objective of this study was to understand the mechanism of FA to decontaminate the paper-mill waste from toxic heavy metals by using *B. napus* plants.

## Materials and Methods

### Study Area Description and Collection of Paper Sludge Samples

**Figure 1:**
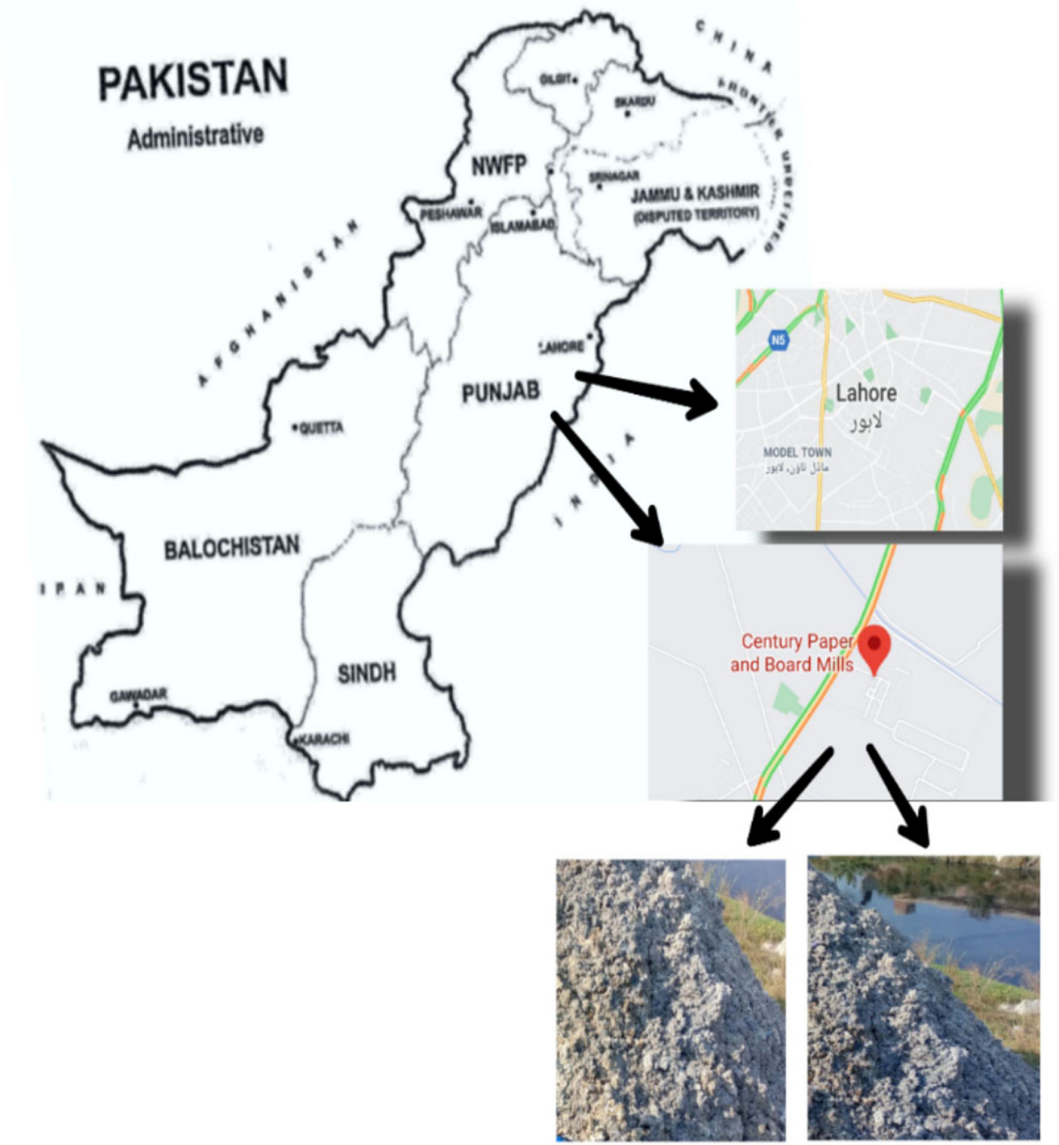
Map of Pakistan showing sampling sites (Century Paper and Board Mill Lahore) and form of paper sludge.

The field survey was carried out in October 2020 at the semi-solid waste dumping site built by the Century Paper and Board Mill (CPBM), which is situated 67 km from Lahore, Punjab, Pakistan (Figure 1). The geographical position of the sampling site was 31°39–511″N 74°08–862″E at an elevation of 669 feet as recorded by GPS (Model: Etrex H Garmin, Taiwan). The soil samples were collected during the survey at a depth of 15 cm. Paper sludge is produced during different operational stages of the paper mill. However, for the current study, a sludge sample was collected at the final operational stage. The sludge was stored in plastic bags and transported to the wire house of the Department of Botany, University of Education, Lahore, Pakistan. The paper sludge samples were dried indoors and sieved using an aluminum sieve (2 mm) to remove impurities, and finally, different concentrations were prepared using distilled water.

### Procurement of *Brassica napus* Seeds and Experimental Setup

Healthy seeds of *Brassica napus* L. were procured from the National Institute for Biotechnology and Genetic Engineering (NIBGE), Faisalabad. The pot experiment was set up [medium-sized plastic pots having a dimension of 10″ × 5″] in the greenhouse. The preliminary experiment was done by using 20, 40, 60, 80, and 100% of paper sludge to find the suitable concentration affecting the growth of *B. napus*. The results indicated that, at 20% of paper sludge, plant growth was restricted, and above 20%, the plants died completely. Thus, four sludge concentrations of 0, 5, 10, and 15% were selected for this investigation to amend garden soil (33% sand, 34% silt, and 33% clay). Soil without sludge contamination was used as a control. Three treatments of fulvic acid at 0, 10, and 20% concentrations were prepared and used alone or in combination with paper sludge by adding directly to the pots. Tap water without FA was used as a control. The experiment consisted of three replicates for each treatment with one plant in each pot. The experiment was laid out following a complete randomized design with a factorial arrangement (Steel and Torrie, 1980) under natural sunlight and temperature (22°C ± 2°C). FA solution was applied to the plants two times a week. The duration of the pot experiment was 90 days.

### Plant Harvest and Analysis of Various Physicochemical and Growth Parameters

Plants were uprooted after 90 days of paper sludge and FA treatment. Different plant parts were separated to measure the growth parameters. Different concentrations of paper sludge were used to determine the pH, electrical conductivity (EC), and total dissolved solids (TDS) by using a meter (HI-9811-5 pH/EC/TDS/°C portable meter) and physicochemical parameters before the experiment was set up for each treatment.

### Estimation of Moisture Content, Ash, Total Organic Carbon, and Volatile Matter

All the above-mentioned parameters were estimated by employing the Merry and Spouncer (1988) method while using the following equations:


(1) $$\begin{array}{rcl} \text{Moisture content}\left(\text{\%}\right) & = & \frac{\text{A}-\text{B}\times 100}{\text{B}} \end{array}$$

(2) $$\begin{array}{rcl} \text{Ash }\left(\text{\%}\right) & = & \frac{\text{C}\times 100}{\text{B}} \end{array}$$

(3) $$\begin{array}{rcl} \text{TOC }\left(\text{\%}\right) & = & 1.703-0.502\;\left(\text{TOM}\right) \end{array}$$

(4) $$\begin{array}{rcl} \text{VM} & = & \left\{\left(\text{A }-\text{ B}/\text{A}\right)\times 100\right\}-\text{M} \end{array}$$

### Determination of Calcium (Ca) and Magnesium (Mg) Content

Calcium and magnesium were calculated by the total hardness described by Eaton et al. (2005). Initially, 10 ml of the sample was taken in the beaker, and ammonia buffer of 2 ml (pH ≈ 10) was added to it. Then, 2 mg Eriochrome black tea (Sigma; EBT) was added as an indicator. The solution was reddish pink in color. This solution was titrated using a standard solution of 0.02N Ethylenediamine tetraacetic acid (EDTA). The color changed from reddish pink to sky blue. The volume of EDTA used was recorded, and finally, total hardness was estimated using the following equation:


(5) $$\begin{array}{r} Total hardness=\frac{Reading of EDTA\times 1000}{Values of a sample used} \end{array}$$

For calcium and magnesium estimation, 10 ml of the sample was taken in the beaker by adding 2 ml of a standard solution of 5N NaOH. Then, 2 mg murexide was added as an indicator until the pink color appeared. This solution was titrated with a standard solution of 0.02N EDTA. When the color changed from pink to purple, the volume of the EDTA used was recorded. The value of calcium and magnesium was calculated using the following equation:


(6) $$\begin{array}{r} Ca-H=\frac{Reading of EDTA\times 1000}{Values of a sample used} \end{array}$$

### Determination of Sodium (Na) and Potassium (K)

The analysis of Na and K was done on Flame Photometer (Janeway PFP-7, UK) following the standard method given in Greenberg et al. (1998).

### Determination of Carbonate and Bicarbonate

The carbonates and bicarbonates were determined by measuring the total alkalinity following the method of Pohland and Bloodgood (1963) and using the following equation:


(7) $$\begin{array}{r} \mathrm{Total}\mathrm{alkalinity}=\frac{\mathrm{Reading}\times 1000}{sample volume} \end{array}$$

### Sample Digestion of Plant and Paper Sludge for Heavy Metals Determination

The different parts of the plants (roots and shoots) were separated, washed, and dried with a blotting paper at room temperature and finally dried in a microwave oven at 80°C for 24 h. Then, the samples were crushed using a pestle and mortar to get a homogeneous mixture. One gram of the plant sample in powdered form was taken in a flask and digested by adding nitric acid (5 ml) and perchloric acid (15 ml) in a ratio of 1:3. Later, these samples were heated on a hot plate till the volume was reduced to half and were cooled down at room temperature. The final volume of the sample was increased to 50 ml by adding distilled water followed by filtration using Whatman filter paper no. 4 (Greenberg et al., 1998). These filtered samples were transferred to 50 ml plastic bottles to determine the concentration of heavy metals.

Five grams of paper sludge samples were taken in the flasks, and to it 75 ml of HNO_3_, 25 ml of H_2_SO_4_, and 50 ml of HClO_4_ in the ratio of 3:1:2 was added. Then, these samples were heated on a hot plate till the volume was reduced to half and allowed to cool. The samples were diluted by adding distilled water to increase the final volume to 100 ml. Afterward, the sample was filtered using Whatman filter paper no. 4 (Greenberg et al., 1998). These filtrated samples were poured into 100 ml plastic bottles and labeled for further determination of heavy metals. All the samples (plant and paper sludge) were analyzed for the concentration of heavy metals by Atomic Absorption Spectrophotometer (GBC, SAVANT AA, Braeside, Australia). Spectrally scientific-grade reagents and Aldrich standard solutions were used in all the chemical analyses, and each analysis was done in triplicate.

### Morphological Parameters and Estimation of Total Chlorophyll Contents

Different morphological parameters like shoot/root length, number of leaves, number of flowers, and fresh/dry weight of root and shoot were measured after completing the experiment. The quantitative estimation of the total chlorophyll from leaves was measured by following the Arnon (1949) method. For this purpose, one gram of fresh leaf sample was crushed into fine powder. This powder was dissolved in 10 ml of acetone solution (80%). The absorbance of this homogenous mixture was measured at 645 nm and 663 nm with the help of a spectrophotometer (T60U UV-Visible UK). The total chlorophyll contents were measured using the following equation:


(8) $$\begin{array}{rcl} \mathrm{Chlorophyll}a & = & \frac{12.7\left(A663\right)-2.69\left(A645\right)\times V}{1000}\times W \end{array}$$

(9) $$\begin{array}{rcl} \mathrm{Chlorophyll}b & = & \frac{22.9\left(A645\right)-4.68\left(A663\right)\times V}{1000}\times W \end{array}$$

(10) $$\begin{array}{rcl} \mathrm{Total}\mathrm{Chlorophyll} & = & \frac{20.2\left(A645\right)-8.02\left(A663\right)\times V}{1000}\times W \end{array}$$

### Determination of Antioxidant Enzymes (Peroxidase, Catalase, and Superoxide Dismutase)

For enzyme assay, one gram of fresh plant material was macerated in liquid nitrogen into a powder form using a pestle and mortar. The pulverized tissue was suspended in 2 ml of 0.1 M phosphate buffer (pH 7.2) with 0.5% (*v/v*). Triton X-100 and 0.1 g of *polyvinyl-pyrrolidone* (PVP) and subjected to centrifugation (Sorval RB-5 refrigerated super-speed) at 12,000 rpm at 4°C for 10 min. The supernatant was used for enzyme assay.

Peroxidases (POD; E.C 1.11.1.7) activity was determined following Luck's (1974) ‘Guaiacol-H_2_O_2_’ method with few modifications. The assay mixture was prepared by adding 3 ml 0.1 M phosphate buffer (pH 7.2), 0.05 ml of 20 mm guaiacol solution, 0.1 ml enzyme extract, and 0.03 ml of 12.3 mm H_2_O_2_ solution. POD activity was calculated by the time required to increase the absorbance by a value of 0.1 (e.g., 0.4–0.5) at 240 nm and expressed in U/ml of the enzyme.

Catalase (CAT; E.C 1.11.1.6) activity was carried out following the Beers and Sizer (1952) method. The reaction was carried out using two buffers (A and B). Buffer A consisted of 50 mm potassium phosphate (pH 7.0), while buffer B was 0.036% H_2_O_2_ solution in 50 mm potassium phosphate solution (pH 7.0). The reaction mixture consisted of 2.9 ml buffer B and 0.1 ml of enzyme extract, while the control consisted of 3 ml of buffer A. The enzyme activity was estimated by the time required for the absorbance (at 240 nm) to decrease from 0.45 to 0.40 and expressed in Uml^−1^ of the enzyme. The catalase activity was calculated by using equation 10.


(11) $$\begin{array}{r} \mathrm{Catalase}\mathrm{activity}\left(\mathrm{units}/\mathrm{ml}\mathrm{enzyme}\right)=\frac{3.45\times \mathrm{df}}{\mathrm{Min}\times 0.1} \end{array}$$

3.45 Corresponds to the Decomposition of 3.45 Micromoles of Hydrogen Peroxide in 3 ml of the Reaction Mixture, Producing a Decrease in the A_240nm_ From 0.45 to 0.40 Absorbance Units.df = dilution factor.Min= Time in minutes required for the A_240nm_ to decrease from 0.45 to 0.40 absorbance units.0.1= Volume of enzyme used (in milliliters).

Superoxide dismutase (SOD; E.C 1.15.1.1) was assayed according to Maral et al. (1977). Two tubes were taken, each containing 2.0 ml of 1.0 mm sodium cyanide (NaCN), 13 mm methionine, 75 μM NBT, 0.1mm EDTA, and 2.0 μM riboflavin as a substrate. One tube was used as a sample containing reaction mixture + 5.0 μL enzyme extract and placed approximately 30 cm below the bank of two 30W fluorescent tubes for 15 min. The other tube containing the reaction mixture without enzyme extract was illuminated simultaneously. The absorbance of the experimental tube was compared to the control at 560 nm. SOD activity was expressed as Umg^−1^ of protein. SOD activity was determined by calculating the percentage inhibition of NBT as equation 12.


(12) $$\begin{array}{r} \text{\%inhibition}\\ \text{=}\frac{\text{Absorbance of control sample - Absorbance of experimental sample}}{\text{Absorbance of experimental sample}}\text{× 100} \end{array}$$

The SOD activity was calculated based on the fact that one unit of SOD caused 50% inhibition.

### Statistical Analyses

CoStat (Cohort Software, Monterey, California) version 6.303 was used to analyze the data. Duncan's Multiple Range Test (DMRT at the significant level of *p* < 0.05) was used to compare the treatment groups (Kim, 2014).

## Results and Discussion

### Physicochemical Parameters of Different Concentrations of Paper Sludge Before Transplantation of *B. napus* Plants

In the present investigation, the physicochemical parameters of paper sludge were studied before the start of the experiment. A comparison (*p* < 0.05) of physicochemical investigation of different concentrations of paper sludge used in this study revealed that all the parameters were highest at 100% concentration than the other concentrations, as shown in Table 1. The highest pH of paper sludge was 8.31 in the 100% PS. While the lowest pH of 7.19 was observed at a 0% concentration of PS. High pH indicates the alkaline nature of paper sludge. These findings are in line with the findings of Abdullah et al. (2015) and Khilji et al. (2021), who reported the variance in the pH (7.45–8.09) of paper sludge from different paper industrial sites. This alkalinity was aggravated by the addition of calcium carbonate (CaCO_3_) utilized during the paper finishing processes. The acidic soil with low pH can be neutralized by CaCO_3_ found in the paper sludge with the help of cellulosic fiber content that has the capacity to hold the moisture content in the soil (Kang et al., 2010). The electrical conductivity (EC) increased as the concentration of PS increased from 5 to100%, and the highest value (475 μScm^−1^) was observed at 100% paper sludge. This was contradictory to Kuokkanen et al. (2008), who observed a lower EC (200 μScm^−1^) value of paper sludge. The nature of the raw material and chemicals used in processing might be the reason for the low EC value. In our research study, TDS was 6,460 mgkg^−1^ in 100% concentration of paper sludge. The highest total dissolved solids in the sample show the presence of inorganic salts (Heydari and Bidgoli, 2012). The high concentration of pH, EC and TDS suggest the toxic conditions for plants which reduced the availability of essential nutrients and growth (Peralta and Costa, 2013). The carbonates were absent in all the concentrations of paper sludge due to their alkaline nature (Simao et al., 2021) while bicarbonates were found highest (4,400 mgkg^−1^) in the 100% concentration of paper sludge and lowest (1,400 mgkg^−1^) in the control with 0% PS. Bicarbonates increased with an increasing concentration of paper sludge. Higher amounts of bicarbonates indicate the high pH of sludge samples tested during this investigation and other studies (Poschenrieder et al., 2018). Among essential nutrients (calcium, magnesium, potassium, sodium, nitrogen, and phosphorus), the maximum (*p* < 0.05) concentration was observed in 100% paper sludge concentration. Magnesium is important for the biosynthesis of chlorophyll pigments and plays a major role in the photosynthesis process (Jadhav et al., 2013). However, a concentration higher than the permissible limit leads to abnormalities in plants such as a reduction in plant growth and yield (Rietra et al., 2017). Similarly, high concentrations of sodium and calcium decreased plant growth and yield by disturbing the photosystems of the plants. However, their high concentration may benefit plants under abiotic stress, especially under salt stress conditions (Chen et al., 2019; Li et al., 2021). The maximum amount of nitrogen, phosphorous, and potassium was observed in the 100% paper sludge, i.e., 350, 200, and 220 mgkg^−1^, respectively. These results confirmed the findings of Martínez et al. (2007) who observed the amount of nitrogen (5,100 mgkg^−1^), phosphorous (3400 mgkg^−1^), and potassium (200 mgkg^−1^) in paper sludge. These are essential elements of the soil but are sometimes available in very fewer amounts to the plants and retard their growth significantly (Khilji and Sajid, 2020). In the case of heavy metals in 100% paper sludge, chromium (680 mgkg^−1^) was observed to be higher than cadmium (55 mgkg^−1^) and lead (400 mgkg^−1^). In this investigation, heavy metals in the paper sludge were found within the permissible limits of National Environment Quality Standard (NEQS) as observed by Fahim et al. (2019). The organic carbon and volatile matter in our study were 23.10 and 13.10%, respectively, in 100% concentration of paper sludge. These results are in line with the findings of Khilji et al. (2021), who also reported a higher percentage of organic carbon (32.75%) and volatile matter (62.3%) in the paper sludge. The moisture content was higher (31.03%) in 100% concentration than 5, 10, 15, and 20% concentrations of PS in the current study. However, this value was far below the findings of Hamzeh et al. (2011), who observed 45%-55% moisture contents due to clay-like nature.

**Table 1:** Physicochemical parameters of different concentrations of paper sludge-amended soil before transplantation of *Brassica napus* plants.

**Parameters**	**Paper sludge concentrations**					
	**0%**	**5%**	**10%**	**15%**	**20%**	**100%**
pH	7.18^c^ ± 0.65	8.14^b^ ± 0.48	8.16^b^ ± 0.59	8.19^b^ ± 0.61	8.2^b^ ± 0.65	8.31^a^ ± 0.66
EC (μS cm^−1^)	69^f^ ±0.70	105^e^ ±0.75	123^d^ ±0.76	165^c^ ±0.70	197^b^ ±0.76	475^a^ ± 0.81
TDS (mg kg^−1^)	938^f^ ± 0.5	1,428^e^ ± 0.60	1,672^d^ ± 0.68	2,244^c^ ± 0.76	2,679^b^ ± 0.85	6,460^a^ ± 0.95
Carbonates (mg kg^−1^)	0	0	0	0	0	0
Bicarbonates (mg kg^−1^)	1,400^f^ ± 0.5	2,000^e^ ± 0.62	2,600^d^ ± 0.76	3,400^c^ ± 0.77	4,000^b^ ± 0.87	4,400^a^ ± 0.90
Calcium (mg kg^−1^)	641^f^ ± 0.55	802^e^ ± 0.60	1,042^d^ ± 0.65	1,283^c^ ± 0.70	1,443^b^ ± 0.75	1,684^a^ ± 0.85
Magnesium (mg kg^−1^)	145^f^ ± 0.60	198^e^ ± 0.68	250^d^ ± 0.76	291^c^ ± 0.95	340^b^s ± 1.00	388^a^ ±1.05
Potassium (mg kg^−1^)	67^f^ ± 0.45	93^e^ ± 0.55	120^d^ ± 0.64	160^c^ ± 0.72	201^b^ ± 0.85	220^a^ ± 0.9
Sodium (mg kg^−1^)	300^f^ ± 0.5	420^e^ ± 0.6	810^d^ ± 0.7	1,020^c^ ± 0.8	1,260^b^ ± 0.85	1,320^a^ ± 0.95
Nitrogen (mg kg^−1^)	75^f^ ± 0.75	110^e^ ± 0.80	150^d^ ± 0.85	200^c^ ± 0.9	290^b^ ± 0.95	350^a^ ± 1.00
Phosphorous (mg kg^−1^)	30^f^ ± 0.50	50^e^ ± 0.55	80^d^ ± 0.60	100^c^ ± 0.65	150^b^ ± 0.70	200^a^ ± 0.75
Organic matter (%)	1.28^f^ ± 0.25	1.77^e^ ± 0.31	2.69^d^ ± 0.12	4.2^c^ ± 0.40	4.89^b^ ± 0.13	12.83^a^ ± 0.67
Cadmium (mg kg^−1^)	0.02^f^ ± 0.001	15^e^ ± 0.60	23^d^ ± 0.56	34^c^ ± 0.61	48^b^ ± 0.70	55^a^ ± 1.00
Chromium (mg kg^−1^)	0.015^f^ ± 0.006	250^e^ ± 0.50	337^d^ ± 0.72	498^c^ ± 0.79	576^b^ ± 0.87	680^a^ ± 1.01
Lead (mg kg^−1^)	0.04^f^ ± 0.005	50^e^ ± 1.12	119^d^ ± 1.13	154^c^ ± 1.20	209^b^ ± 1.28	400^a^ ± 2.5
Moisture content (%)	8.29^f^ ±0.20	9.45^e^ ±0.24	10.36^d^ ±0.25	14.15^c^ ±0.30	15.32^b^ ±0.35	31.03^a^ ±0.42
Ash (%)	97.67^a^ ± 0.31	96.81^b^ ± 0.35	95.15^c^ ± 0.36	92.44^d^ ± 0.42	91.19^e^ ± 0.47	76.89^f^ ± 0.48
Organic matter (%)	2.32^f^ ± 0.16	3.19^e^ ± 0.31	4.85^d^ ± 0.36	7.56^c^ ± 0.42	8.81^b^ ± 0.45	23.10^a^ ± 0.55
Volatile matter (%)	81.41^a^ ± 0.45	75.88^b^ ± 0.48	74.78^c^ ± 0.49	63.62^d^ ± 0.56	69.36^e^ ± 0.65	13.10^f^ ± 0.70

*Each value is a mean ± standard deviation of 3 replicates, and different superscript letters in each column show significant differences according to DMRT at p ≤ 0.05*.

*FA, Fulvic acid; PS, Paper sludge*.

### Morphological Parameters of *B. napus* Plants Irrigated With Fulvic Acid Grown at Different Concentrations of Paper Sludge

**Figure 2:**
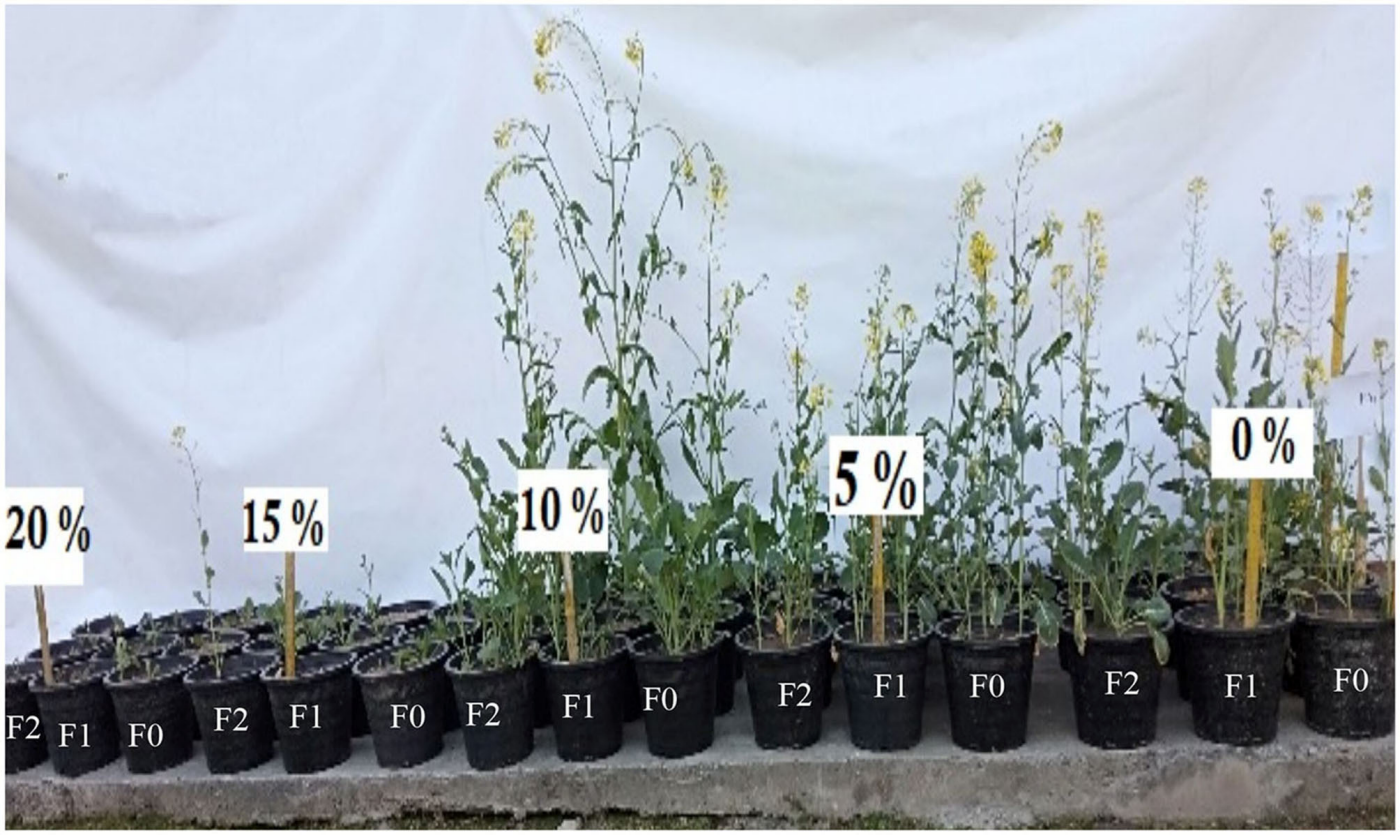
A complete view of the experiment at various concentrations of PS (0–15%) and FA (0, 10, and 20%; F0, F1, and F2) at the time of harvesting.

**Figure 3:**
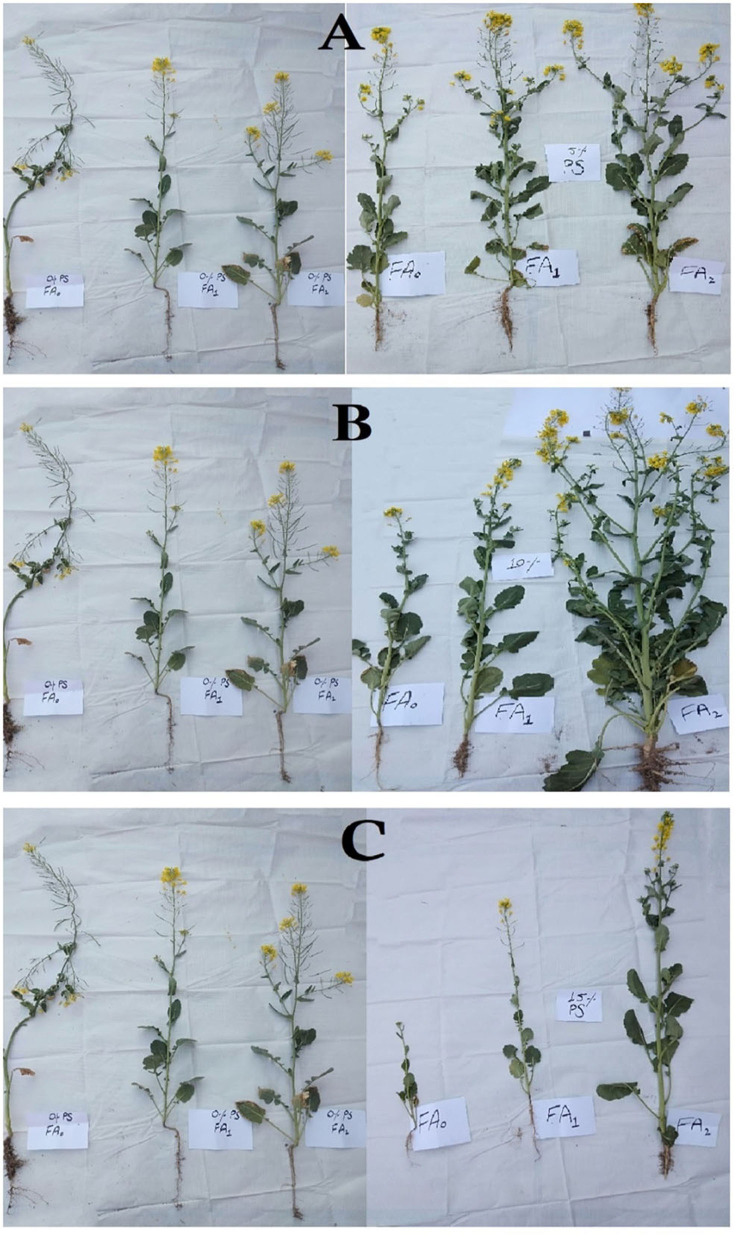
**(A)** Comparison of control (0%) with 5% concentration of paper sludge. **(B)** Comparison of control with 10% concentration of paper sludge. **(C)** Comparison of control with 15% concentration of paper sludge.

It is evident from the literature review that heavy metals cause toxic effects in plants by reducing growth and ultimately leading to their death by impeding various physiological mechanisms of plants (Singh et al., 2016; Prerna et al., 2019; Mubeen et al., 2022). These heavy metals compete with nutrient cations for adsorption, alter the structure and function of plants, produce reactive oxygen species, and disrupt the functions of enzymes (Chaab et al., 2015; Ahmad et al., 2021; Faiz et al., 2022). Various concentrations (0, 10, and 20%) of FA were applied to *B. napus* plants grown in soils amended with different concentrations of paper sludge to modify the chemical nature and bioavailability of heavy metals during this investigation. Results indicated that all the morphological parameters (shoot/root length, fresh/dry weight, number of leaves, and flowers) reduced significantly (*p* < 0.05) in plants subjected to 15% concentration of PS (Figure 2). This might be due to the increased accumulation of toxic metals by plants that caused metal stress for the growth of *B. napus* plants. Higher concentrations of heavy metals reduced growth in plants, which has been investigated in previous studies (Pant and Tripathi, 2014; Khilji and Sajid, 2020). In the case of shoot/root length, a significant decrease in fresh/dry weight of shoot/root was observed at 15% PS (Table 2, Figures 3A–C). However, the application of FA alleviated metal toxicity. The results of morphological parameters indicated that the growth of plants grown at various concentrations of PS significantly (*p* < 0.05) increased by FA supplementation. Shoot length was enhanced to 31.66 cm, 26 cm, and 14.05 cm from 27.33, 17.83, and 7.5 cm by applying 20% concentration of FA to plants grown at 5, 10, and 15% concentration of PS, respectively. Similarly, 20% treatment of FA enhanced the fresh/dry weight of the shoot from 14.55, 10.05, and 3.36 g/2.20 g, 2.74 g, 0.48 g to 18.8 g, 13.99 g, 6.72 g/6.84 g, 5.33 g, 5.05 g at 5, 10 and 15% of PS. The exposure of plants to 5, 10, and 15% concentrations of PS prominently (*p* < 0.05) reduced the number of leaves and flowers. When these PS-grown plants were treated with 20% FA, the number of leaves and flowers increased from 5.86 and 2 to 8.13 and 2.3 at 15% of PS, respectively. Fulvic acid not only enhances the availability of essential nutrients but modifies the nature of heavy metal and reduces uptake of heavy metals in the plants (Yang et al., 2013). It has a high cation exchange capacity and forms strong bonds with heavy metal, thereby reducing the stress in plants (Ozkay et al., 2016). These results were in line with Esringü et al. (2021), who observed maximum fresh and dry weight in shoot/root and maximum number of leaves and flowers in the *Impatiens walleriana* L. when treated with FA. According to Esringü et al. (2021), the growth, yield, and dry matter of *Tagetes eracta* and *Zinnia elegans* Jacq reduced as a result of maximum accumulation of heavy metals without FA treatment. It was observed that, by increasing the FA concentration gradually, all the growth parameters increased significantly. These results confirm previous findings of Abdel-Bakey et al. (2019), who observed that the faba bean (*Vicia faba* L.) showed maximum growth at a higher concentration of fulvic acid. Our findings are also in line with Moradi et al. (2017), who observed a high yield of safflower by treating it with fulvic acid.

**Table 2:** Comparison of morphological parameters of *Brassica napus* L. irrigated with fulvic acid grown at different concentrations of paper sludge.

**Growth parameters**	**Concentrations of paper sludge (%)**	**Treatments of FA**		
		**0**	**10%**	**20%**
Root length (cm)	0	4.0^cC^ ± 1.56	6.16^bcB^ ± 2.86	6.7^cA^ ± 2.52
	5	6.0^bB^ ± 1.73	6.66^bA^ ± 2.51	7.0^bA^ ± 2.53
	10	6.83^aC^ ± 0.76	7.33^aB^ ± 1.52	8.0^aA^ ± 1.00
	15	3.0^dB^ ± 1.89	3.8^cA^ ± 2.37	3.93^dA^ ± 2.67
Shoot length (cm)	0	24.33^bB^ ± 2.69	28.6^aA^ ± 2.77	29^aA^ ± 2.64
	5	27.33^aB^ ± 2.79	28.16^aB^ ± 2.67	31.66^aA^ ± 2.78
	10	17.83^cC^ ± 2.14	20.0^bB^ ± 2.01	26^bA^ ± 2.77
	15	7.5^dB^ ± 1.23	13.0^cA^ ± 1.01	14.5^cA^ ± 1.52
Number of leaves	0	9.33^aB^ ± 1.05	12.33^aA^ ± 2.21	13.33^aA^ ± 2.88
	5	9.66^aC^ ± 1.30	11.0^bB^ ± 2.81	13^aA^ ± 2.55
	10	7.66^bB^ ± 1.86	9.66^cA^ ± 2.42	9.93^cA^ ± 2.82
	15	5.86^cB^ ± 0.80	8.0^dA^ ± 2.57	8.13^cA^ ± 2.08
Number of flowers	0	2.33^aB^ ± 1.20	2.66^aB^ ± 1.30	3.0^aA^ ± 2.10
	5	2.66^aA^ ± 2.30	2.99^aA^ ± 1.40	3.06^aA^ ± 1.99
	10	2.0^bB^ ± 0.15	2.33^bB^ ± 2.11	3.1^aA^ ± 1.54
	15	2.0^bB^ ± 0.05	2.5^bA^ ± 0.5	2.0^bB^ ± 1.00
Fresh weight of shoot (g)	0	15.42^aC^ ± 1.04	16.03^aB^ ± 2.09	18.86^aA^ ± 2 0.38
	5	14.55^aB^ ± 1.02	14.94^bB^ ± 2.40	18.8^aA^ ± 1.12
	10	10.05^bC^ ± 1.27	12.93^cB^ ±1.85	13.99^aA^ ± 1.49
	15	3.36^cC^ ± 1.94	5.18^dB^ ± 1.47	6.72^cA^ ± 1.47
Fresh weight of root (g)	0	2.39^aB^ ± 0.27	2.90^aA^ ± 1.03	3.00^aA^ ± 1.10
	5	2.13^aB^ ± 0.64	2.68^aA^ ± 1.09	2.98^bA^ ± 2.57
	10	1.77^bC^ ± 0.85	2.05^bB^ ± 0.31	2.83^bA^ ± 1.38
	15	0.64^cC^ ± 0.11	1.59^cB^ ± 0.13	1.92^cA^ ± 1.73
Dry weight of shoot (g)	0	2.49^aB^ ± 1.10	2.87^bB^ ± 0.76	6.84^aA^ ±1.75
	5	2.20^aB^ ± 0.50	2.77^bB^ ± 0.64	5.33^bA^ ± 1.61
	10	2.74^aC^ ± 0.60	3.53^aB^ ± 0.28	5.05^bA^ ±1.33
	15	0.48^bB^ ± 0.10	0.53^cA^ ± 0.20	0.59^cA^ ± 0.60
Dry weight of root (g)	0	0.68^aB^ ± 0.20	0.93^aA^ ± 0.96	1.04^aA^ ± 0.23
	5	0.46^bC^ ± 0.85	0.80^aB^ ± 0.03	0.96^aA^ ± 0.96
	10	0.39^cC^ ± 0.77	0.65^bB^ ± 0.66	0.80^aA^ ± 0.41
	15	0.31^cC^ ± 0.17	0.64^bA^ ± 0.02	0.69^cA^ ± 0.50

*Each value is a mean ± standard deviation of 3 replicates, and different superscript lowercase letters in each column and capital letters within rows show a significant difference. Values with similar letters are significantly not different from each other according to DMRT at p ≤ 0.05*.

*FA, Fulvic acid; PS, Paper sludge*.

### Chlorophyll Content at Various Treatments of FA of *B. napus* Grown at Different Concentrations of Soils Amended With Paper Sludge

**Figure 4:**
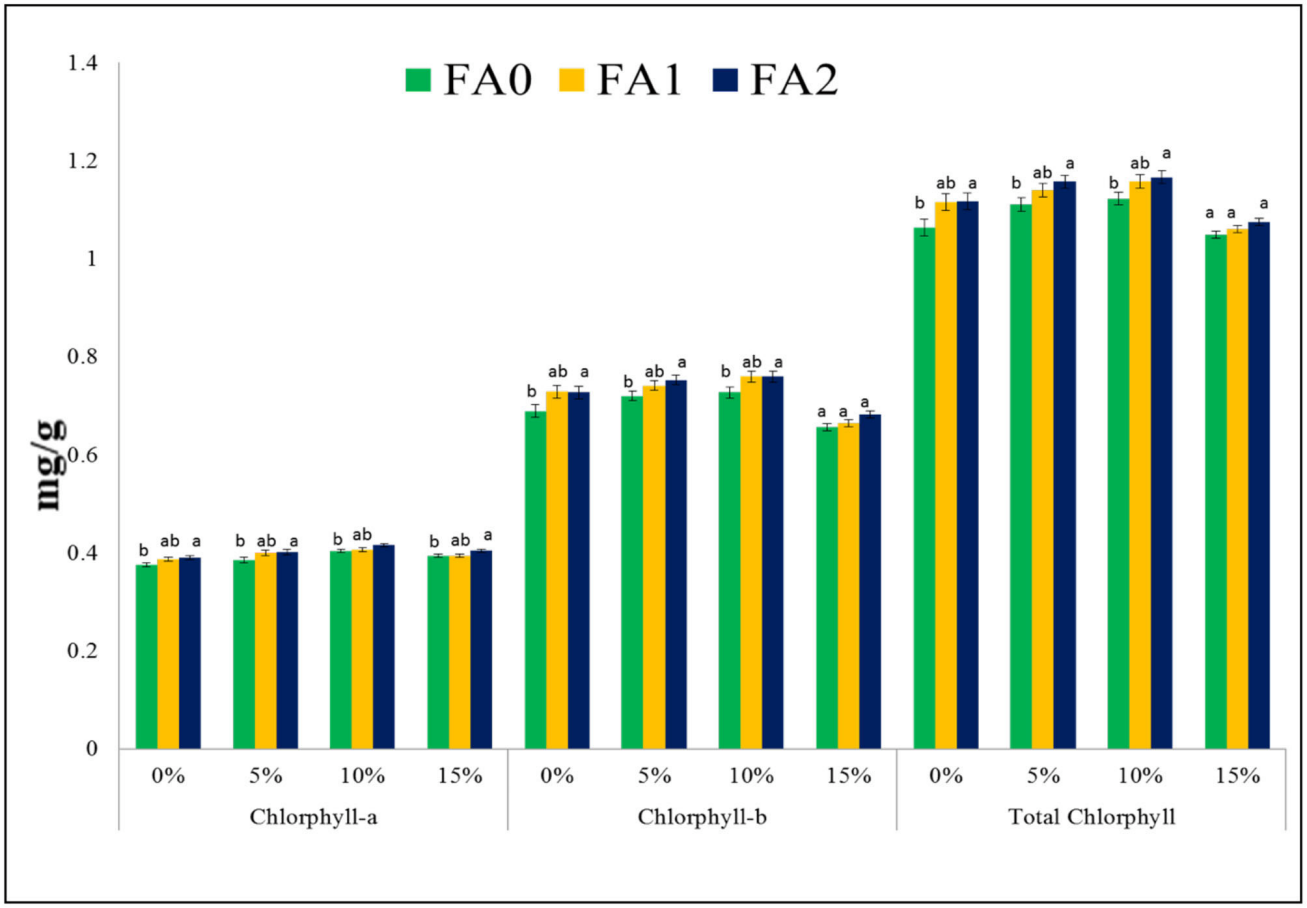
Chlorophyll content at various treatment levels of FA (0, 10, and 20%; F0, F1, and F2) of *Brassica napus* L. grown at different concentrations of soil amended with paper sludge.

The amount of chlorophyll increased (*p* < 0.05) in order of 0 < 5 < 10% of paper sludge concentration with 5, 10, and 20% application of FA to *B. napus* plants in the present investigation. The chlorophyll content was higher in the absence of PS and decreased when the concentration of paper sludge increased. However, with increase in the concentration of FA in all treatments of PS, the chlorophyll content increased gradually (Figure 4). A maximum chlorophyll content was observed in the 10% concentration of PS than in 0 and 5% concentrations at all FA treatments. Increasing quantities of chlorophyll in the paper sludge might be due to the availability of essential nutrients to the plants. In addition to this, FA plays a role in metal stabilization by forming metal complexes and thereby reducing their mobility and toxicity in leaves. According to Lotfi et al. (2015), fulvic acid shows a positive effect on the plants with regards to chlorophyll content by inhibiting the synthesis of reactive oxygen species and enhancing the antioxidant activities of the enzymes under stress conditions. A decrease in chlorophyll content was observed at 15% concentration of PS with FA treatment. Earlier reports have also indicated this reduction in chlorophyll content at higher concentrations of heavy metals due to their toxicity (Mishra et al., 2006). Until now, there is no cost-effective way to remove heavy metals from the environment (Shahzadi et al., 2021) or to inhibit their transfer; mitigation techniques are the only efficient way to alleviate heavy metal stress.

### Antioxidant Enzyme Activities of *B. napus* Plants Grown at Different Concentrations of Paper Sludge

Antioxidant enzyme activities were decreased significantly by increasing the sludge concentration from 0 to 15%. However, by applying FA to plants, these enzymes showed an increasing trend on all tested treatments of PS. Peroxidase activity was highest (5.31, 5.42, 4.20, and 3.93 Uml^−1^ of enzyme at 0, 5, 10 and 15% PS) at 20% concentration of FA. Similar positive effect of FA was also observed in catalase and superoxide dismutase activities in plants growing on various treatments of paper sludge. Catalase activity was increased from 2.08 to 3.11 and 3.93 Uml^−1^ of enzyme at 10 and 20% concentrations of FA. Superoxide dismutase activity was also increased by increasing FA concentrations. SOD activity was increased from 13.33 to 14.33 Umg^−1^, and 14.93 Umg^−1^ of protein at 10 and 15% PS (Table 3). FA has been reported as an antioxidant stimulant under stress in several previous studies (Lotfi et al., 2015; Munir et al., 2021; Yildirim et al., 2021). This increase in antioxidant enzyme activities indicates that FA might be directly involved to mitigate the oxidative stress or act as a signal molecule to enhance the production of antioxidant enzymes, which in turn is reflected in better growth parameters of the plants under extreme environmental stress conditions.

**Table 3:** Determination of antioxidant enzyme of *Brassica napus* L. plants grown at different concentrations of paper sludge after 90 days of the experiment.

**Antioxidant Enzyme activity**	**Concentrations of paper sludge (%)**	**Treatments of Fulvic acid**		
		**0**	**10%**	**20%**
POD Activity (UmL^−1^ of enzyme)	0	4.74^aC^ ± 1.56	5.26^aB^ ± 0.86	5.91^aA^ ± 1.02
	5	4.16^bB^ ± 1.73	4.06^bB^ ± 1.21	5.42^aA^ ± 1.53
	10	3.83^cB^ ± 0.76	3.93^cB^ ± 1.02	4.60^bA^ ± 1.00
	15	2.08^dC^ ± 0.89	3.11^dB^ ± 1.07	3.93^cA^ ± 1.67
CAT Activity (UmL^−1^ of enzyme)	0	14.33^aC^ ± 1.09	15.6^aB^± 1.77	16.19^aA^ ± 1.64
	5	10.33^bC^ ± 1.79	11.06^bB^ ± 2.07	11.66^bA^± 1.78
	10	9.03^cB^ ± 1.14	9.30^cB^± 1.01	10.16^cA^ ± 1.77
	15	7.25^dB^± 1.23	8.10^dA^± 1.01	8.91^dA^ ± 1.52
SOD Activity (Umg^−1^ of Protein)	0	13.33^aB^ ± 1.05	14.33^aA^ ± 2.21	14.93^aA^ ± 0.88
	5	9.66^aC^ ± 1.10	11.0^bB^ ± 2.81	13.2^bA^± 1.05
	10	7.66^cC^ ± 1.06	9.06^cB^± 1.42	9.93^cA^ ± 0.82
	15	5.86^dB^ ± 0.81	8.0^dA^± 1.07	8.13^dA^ ± 0.08

*Each value is a mean ± standard deviation of 3 replicates, and different superscript lowercase letters in each column and capital letters within rows show significant differences. Values with similar letters are significantly not different from each other according to DMRT at p ≤ 0.05*.

### Cr, Cd, and Pb Contents of *B. napus* Plants Grown at Different Concentrations of Paper Sludge

The determination of Cr, Cd, and Pb in the root and the shoot of *Brassica* plants was studied 90 days after the experiment (Table 4). A change in the behavior of metal uptake by roots and shoots was observed in the case of sludge-treated plants after FA application. As the concentration of paper sludge was increased from 0 to 15%, the content of tested heavy metals increased gradually in roots and shoots. On the other hand, FA application (all treatments) reduced the metal uptake in plants at all PS concentrations. Minimum (*p* < 0.05) Cd, Cr, and Pb uptake was observed at 0% treatment of paper sludge in shoots and roots, while the maximum Cd, Cr, and Pb content was observed at 15% PS concentration. Results showed that Cr, Cd, and Pb content was 0.004, 0.004, and 0.019 mgkg^−1^, respectively, in shoots at 0% PS. In roots, these metals were in a range of 0.15, 0.05, and 0.03 mgkg^−1^. On the other hand, FA application at 20% concentration reduced Cr, Cd, and Pb uptake in the shoots from 6.08, 34.42, and 20.6–3.62, 17.33, and 15.22 mgkg^−1^, respectively. At this concentration of paper sludge in the root, 20% FA reduced Cr, Cd, and Pb uptake from 11.19, 44.11, and 35.5–7.88, 27.01, and 24.02 mgkg^−1^, respectively. These results are similar to the findings of Ali et al. (2015), who observed that fulvic acid enhances Cr tolerance in *Triticum aestivum* L. by reducing Cr uptake and improving the antioxidant defense system of applied plants. In this study, *B. napus* plants accumulated a higher amount of Cr, Cd, and Pb in the roots than in the shoots. This finding is in line with Shah et al. (2020), who observed that lead accumulates in higher amounts in roots than in shoots of *Brassica* plants. Similarly, the maximum amount of Cd and Cr was accumulated in the roots of canola and garden cress in this study as well as earlier studies (Yu et al., 2012; Yildirim et al., 2021). In the current study, it was observed that Cr, Cd, and Pb accumulated within the permissible limits of NEQS (0.1, 0.1, and 0.5 mgL^−1^, respectively) in the shoots at a 10% concentration of paper sludge. However, at a 15% concentration of paper sludge, their accumulation was above this limit, which resulted in retarding the plant growth and decreasing the number of leaves and flowers during the present investigation. At higher concentrations of paper sludge, the adverse effect of heavy metals was also observed in several plant species in earlier studies (Arif et al., 2016; Asati et al., 2016; Goyal et al., 2020).

**Table 4:** Determination of Cr, Cd, and Pb contents of *B. napus* plants grown at different concentrations of paper sludge after 90 days of the experiment.

**Metal contents (mgkg^**−1**^)**			**Treatments of FA**		
	**Plant part**	**Con**.	**0**	**10%**	**20%**
**Cadmium**	Root	0%	0.15^a^ ± 0.05	0.01^c^ ± 0.005	0.05^b^ ± 0.007
	Shoot	0%	0.004^a^ ± 0.01	0.002^ab^ ± 0.002	0.001^b^ ± 0.002
	Root	5%	6.11^a^ ± 0.49	5.05^b^ ± 0.13	3.23^c^ ± 0.11
	Shoot	5%	2.07^a^ ± 0.31	1.97^b^ ± 0.14	1.23^c^ ± 0.12
	Root	10%	7.03^a^ ± 0.35	5.05^b^ ± 0.16	3.98^c^ ± 0.09
	Shoot	10%	2.81^a^ ± 0.07	2.03^b^ ± 0.06	1.09^c^ ± 0.08
	Root	15%	11.19^a^ ± 0.14	8.45^b^ ± 0.05	7.88^c^ ± 0.04
	Shoot	15%	6.08^a^ ± 0.20	5.87^b^ ± 0.08	3.62^c^ ± 0.06
**Chromium**	Root	0%	0.05^a^ ± 0.01	0.02^ab^ ± 0.007	0.01^b^ ± 0.006
	Shoot	0%	0.004^a^ ± 0.01	0.002^ab^ ± 0.003	0.001^b^ ± 0.001
	Root	5%	8.72^a^ ± 0.06	7.99^b^ ± 0.05	5.75^c^ ± 0.03
	Shoot	5%	5.61^a^ ± 0.32	3.88^b^ ± 0.09	2.07^c^ ± 0.08
	Root	10%	6.92^a^ ± 0.09	6.03^b^ ± 0.06	4.80^c^ ± 0.05
	Shoot	10%	4.04^a^ ± 0.24	2.11^b^ ± 0.10	1.06^c^ ± 0.08
	Root	15%	44.11^a^ ± 0.10	38.09^b^ ± 0.09	27.01^c^ ± 0.06
	Shoot	15%	34.42^a^ ± 0.27	22.34^b^ ± 0.23	17.33^c^ ± 0.22
**Lead**	Root	0%	0.03^a^ ± 0.01	0.02^b^ ± 0.007	0.01^b^ ± 0.02
	Shoot	0%	0.019^a^ ± 0.04	0.001^b^ ± 0.001	0.002^b^ ± 0.001
	Root	5%	10.65^a^ ± 0.06	8.66^b^ ± 0.05	5.77^c^ ± 0.04
	Shoot	5%	6.37^a^ ± 0.08	5.67^b^ ± 0.05	2.55^c^ ± 0.04
	Root	10%	8.05^a^ ± 0.47	7.06^b^ ± 0.36	4.11^c^ ± 0.26
	Shoot	10%	5.04^a^ ± 0.12	2.99^b^ ± 0.07	1.01^c^ ± 0.06
	Root	15%	35.15^a^ ± 0.48	29.88^b^ ± 0.14	24.02^c^ ± 0.12
	Shoot	15%	20.6^a^ ± 0.10	18.44^b^ ± 0.07	15.22^c^ ± 0.06

*Each value is a mean ± standard deviation of 3 replicates, and different superscript letters in each column show significant differences according to DMRT at p ≤ 0.05*.

*FA, Fulvic acid; PS, Paper sludge*.

### Post-harvest Analysis of Physicochemical Parameters of Different Concentrations of Paper Sludge After Harvesting

After harvesting the plants on day 90 of FA and PS treatment, the soils were preserved for the post-harvesting analysis of physicochemical parameters (Table 5). The analysis indicated that the amount of all tested metals (trace and toxic) was reduced in all concentrations of PS after transplantation. After stabilizing the toxic heavy metals, the paper sludge was considered a safe material to be added to the soil for ground reclamation and soil improvement. Similar findings were observed by Akcin and Akcin (2019) and Antonkiewicz et al. (2018), who observed that Cr concentration was reduced in the paper sludge-amended soil after harvesting the plants. The underlying reason for this reduction in heavy metals might be the formation of metal-FA complexes (Tang-Wang et al., 2014). These complexes are formed due to the presence of many carboxyl and hydroxyl groups. Moreover, fulvic acid efficiently immobilizes the toxic metals in the soil due to its smaller size and higher oxygen content. FA has been reported to inhibit the accumulation of metals by reducing its availability to plants and is considered very effective for metal bioremediation in alkaline soils (Park et al., 2012). This phytostabilization of toxic heavy metals by FA application of canola plants is effective in reclaiming and improving the paper sludge-contaminated soils for better agriculture.

**Table 5:** Postharvest analysis of physicochemical parameters of different concentrations of paper sludge after plant harvesting.

**Parameters**	**Paper sludge concentrations**			
	**0%**	**5%**	**10%**	**15%**
pH	7.9^a^ ± 0.05	8.0^a^ ± 0.52	8.07^a^ ± 0 0.62	8.12^a^ ± 1.07
EC (μScm^−1^)	59^d^ ± 0.55	99^c^ ± 0.65	110^b^ ± 0.95	145^a^ ± 1.00
TDS (mgkg^−1^)	802^d^ ± 0.62	1,346^c^ ± 0.73	1,496^b^ ± 0.89	1,972^a^ ± 1.01
Carbonates (mgkg^−1^)	0	0	0	0
Bicarbonates (mgkg^−1^)	1,250^d^ ± 0.95	1,569 ^c^± 0.97	1,890^b^ ± 0.98	2,589^a^ ± 0.99
Calcium (mgkg^−1^)	467^d^ ± 0.24	645^c^ ± 0.55	890^b^ ± 0.80	1,032^a^ ± 0.90
Magnesium (mgkg^−1^)	95^d^ ± 0.90	112^c^ ± 0.97	149^b^ ± 0.98	199^a^ ± 0.99
Potassium (mgkg^−1^)	59^d^ ± 0.55	63^c^ ± 0.61	84^b^ ± 0.79	98^a^ ± 0.85
Sodium (mgkg^−1^)	149^d^ ± 0.60	230^c^ ± 0.64	461^b^ ± 0.72	690^a^ ± 1.00
Nitrogen (mgkg^−1^)	35^d^ ± 0.70	67^c^ ± 0.80	74^b^ ± 0.85	89^a^ ± 1.01
Phosphorous (mgkg^−1^)	14^d^ ± 0 0.67	20^c^ ± 0.81	39^b^ ± 1.31	51^a^ ± 1.58
Organic carbon (%)	0.99^c^ ± 0.07	1.00^c^ ± 0.10	1.50^b^ ± 0.20	1.90^a^ ± 0.50
Cadmium (mgkg^−1^)	0.0015^d^ ± 0.01	10^c^ ± 0.60	15^b^ ± 0.89	22^a^ ± 0.93
Chromium (mgkg^−1^)	0.001^d^ ± 0.001	180^c^ ± 0.68	280^b^ ± 0.71	301^a^ ± 0.76
Lead (mgkg^−1^)	0.002^d^ ± 0.001	22^c^ ± 0.67	80^b^ ± 0.82	105^a^ ± 1.00

*Each value is a mean ± standard deviation of 3 replicates, and different superscript letters in each column show significant differences according to DMRT at p ≤ 0.05*.

*0% = Control (Soil); PS, Paper Sludge*.

## Conclusion

This research revealed that phytostabilization is ideal for reducing heavy metals, especially at industrial waste management sites. *Brassica napus* L. is an efficient plant for the accumulation of all toxic metals, such as Cr, Cd, and Pb. Fulvic acid treatment increased the essential nutrient uptake and minimized the effect of toxic metals by modifying/improving the physical and chemical nature of soils and metals. FA also enhanced the activities of antioxidant enzymes (POD, CAT, and SOD), which in turn overcame the oxidative stress produced under heavy metal stress. In this way, FA not only mitigated the impact of toxic metals from contaminated soils but also enhanced the growth and productivity of hyperaccumulator plants. This phytotechnology is best suited for industries to utilize their waste because it is cheap and more eco-friendly than all the other technologies used for waste management. The positive effect of FA evidenced in this study necessitates further research to evaluate and harness the potential benefits of FA.

## Data Availability Statement

The original contributions presented in the study are included in the article/supplementary material, further inquiries can be directed to the corresponding author/s.

## Author Contributions

SAK designed the study. SF performed the experiments. ZAS and AAS made the chemical analysis and revised the English composition. ANS, SF, and MR analyzed the experiment data. SHY, MA, and ZAS proofread the manuscript. All authors discussed the results and commented on the manuscript. All authors have read and agreed to the published version of the manuscript.

## Funding

The publication of the present work is supported by the Natural Science Basic Research Program of Shaanxi Province (Grant No. 2018JQ5218), the National Natural Science Foundation of China (51809224), the National Research Foundation of Korea (NRF) grant funded by the Korea government (MSIT) (NRF-2021R1F1A1055482), and the Top Young Talents of Shaanxi Special Support Program.

## Conflict of Interest

The authors declare that the research was conducted in the absence of any commercial or financial relationships that could be construed as a potential conflict of interest.

## Publisher's Note

All claims expressed in this article are solely those of the authors and do not necessarily represent those of their affiliated organizations, or those of the publisher, the editors and the reviewers. Any product that may be evaluated in this article, or claim that may be made by its manufacturer, is not guaranteed or endorsed by the publisher.
